# Harnessing of Diluted Methane Emissions by Direct Partial Oxidation of Methane to Methanol over Cu/Mordenite

**DOI:** 10.1021/acs.iecr.1c01069

**Published:** 2021-06-24

**Authors:** Mauro Álvarez, Pablo Marín, Salvador Ordóñez

**Affiliations:** Catalysis, Reactors and Control Research Group (CRC), Department of Chemical and Environmental Engineering, University of Oviedo, Faculty of Chemistry, Julián Clavería 8, 33006 Oviedo, Spain

## Abstract

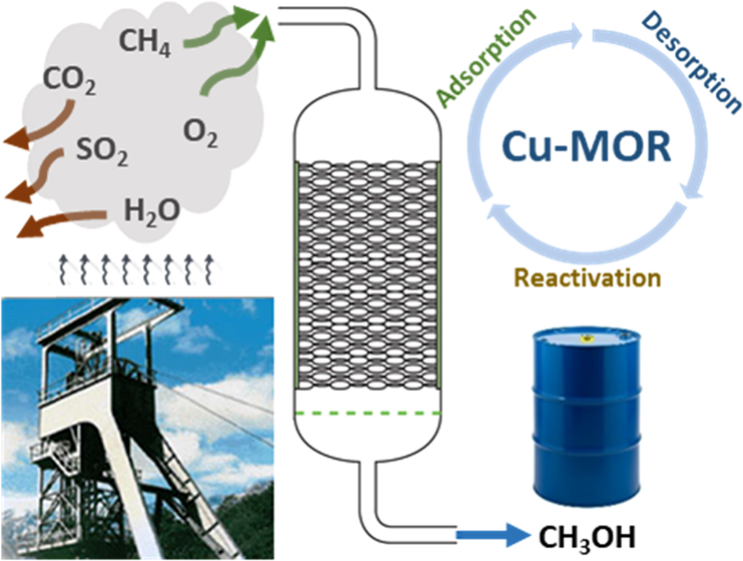

The upgrading of diluted methane emissions into valuable products can be accomplished at low temperatures (200 °C) by the direct partial oxidation of methanol over copper-exchanged zeolite catalysts. The reaction has been studied in a continuous fixed-bed reactor loaded with a Cu–mordenite catalyst, according to a three-step cyclic process: adsorption of methane, desorption of methanol, and reactivation of the catalyst. The purpose of the work is the use of methane emissions as feedstocks, which is challenging due to their low methane concentration and the presence of oxygen. Methane concentration had a marked influence on methane adsorption and methanol production (decreased from 164 μmol/g Cu for pure methane to 19 μmol/g Cu for 5% methane). The presence of oxygen, even in low concentrations (2.5%), reduced methane adsorption drastically. However, methanol production was only affected slightly (average decrease of 9%), concluding that methane adsorbed on the active centers yielding methanol is not influenced by oxygen.

## Introduction

The atmospheric concentration of greenhouse gases (GHGs), responsible for global climate change, has risen steadily in the last few decades.^1^ Nowadays, the focus is on CO_2_ emission reduction; however, methane is also a major contributor to global warming, constituting around 20% of the total GHG emissions.^2,3^ Methane global warming potential (GWP) is 28 times higher than that of CO_2_ (100 year period).^4,5^ Many sectors are responsible for anthropogenic methane emissions, such as agriculture, waste management, oil and gas industry, or coal mining.^6,7^ Many of these emissions are characterized by a small methane concentration, along with high volumetric flow rates. Other compounds, such as water vapor, oxygen, solid particles, or sulfur and nitrogen compounds, are often present in these emissions. For this reason, the harnessing of these emissions as a methane feedstock is a challenging task.^8^ Some authors have proposed the application of combustion technologies to transform methane into CO_2_, which has a lower GWP, and recover some energy (i.e., power or heat).^9,10^ For example, the use of thermal or catalytic afterburners in coal mines for the abatement of ventilation air methane, representing 8% of methane worldwide emissions, can reduce the carbon footprint considerably.^8,11^

However, it is more interesting to transform these methane emissions into value-added products. Methanol is a well-known and versatile platform molecule, widely used by the industry as a chemical or fuel.^12,13^ This transformation would simplify its transportation and storage by increasing its energy density.^5^ The most spread technology used for methanol production consists of a two-step process that uses natural gas as feedstock: first, methane is transformed into syngas via steam reforming and then the syngas is converted into methanol. This process has high capital and energy requirements, so its implementation is not profitable in many scenarios, particularly when lean methane emissions are used as feedstocks.^14−17^ For this reason, the search for a cheaper and straightforward process to convert methane into methanol by partial oxidation has been an aim of the scientific community in the last decades.^18−21^

Methanotrophic bacteria can transform methane into methanol at soft conditions using monooxygenase enzymes.^14,22^ Considering a future industrial implementation, the use of heterogeneous catalysts is a better option. Different materials have been investigated to reproduce the behavior of monooxygenase enzymes. Zeolites, an aluminosilicate material formed by parallel and regular channels with a highly ordered internal structure, have been studied for years with different applications as adsorbents and catalysts. It has recently been discovered that they can also host active metal sites that mimic those on methane monooxygenase enzymes, which can activate the methane C–H bond at low temperatures.^23−26^ This activation is caused by the combination of the catalytic behavior of copper metal and the confinement effects of the zeolite structure.^27^ Many authors have studied zeolites and their different topologies for this reaction, such as ZSM5^26,28,29^ or SSZ13.^30−32^ However, catalysts based on mordenite (MOR) zeolites are the ones with the best performance. This effect is attributed to their large pores, which facilitate product desorption, and the presence of 8MR side pockets, suitable to locate extra-framework copper cations.^27,33−35^

The formation of the active sites within the zeolite structure, the configuration of the active centers, and the reaction mechanism are still under discussion by the scientific community.^36,37^ Some authors proposed a binuclear bis(μ-oxo)dicopper ion ([Cu(μ-O)2Cu]^2+^) as the active site.^38,39^ However, other studies proposed the presence of mono(μ-oxo)dicopper ions ([Cu(μ-O)Cu]^2+^),^40^ trinuclear ions ([Cu_3_(μ-O)_3_]^2+^),^41,42^ or even the simultaneous presence of these species in the zeolite.^43^ At low temperatures, these active sites can activate the C–H bond on methane, leading to an intermediate methoxy species, which are strongly adsorbed. However, if the temperature is too high, these intermediates will be oxidized to carbon oxides.^44^ Therefore, to desorb methanol from the active sites, the temperature cannot be increased. Instead, water, as a liquid^44^ or vapor,^25^ is used to decrease the energy required for methanol desorption using water coadsorption.^13^ Nonetheless, the role of water in this step is also under discussion; some authors propose that water can also stabilize the reaction intermediates.^45^

The overall process of methane oxidation to methanol in these zeolites consists of a three-step chemical looping process. First, the catalyst is activated at high temperatures (450 °C) in the presence of oxidant species (e.g., oxygen). Then, methane is introduced and adsorbed on the active centers at low temperatures (around 200 °C). Finally, methanol is desorbed from the catalyst surface at low temperature using a sweep gas containing water.^46,47^ After this last step, water must be desorbed from the catalyst and the active centers reactivated at high temperatures.

Most of the works from the literature use pure methane as feedstock. However, the present work is focused on the use of lean methane emissions. These feedstocks are difficult to harness by conventional technologies due to their low concentration or the presence of other compounds, like oxygen. It is unknown how the catalysts used in the direct partial oxidation of methane to methanol would perform at these conditions.

The present work aims to fill the gap in this field and elucidate whether this technology can be effectively applied to lean methane feedstocks. To accomplish this goal, a copper–mordenite catalyst has been prepared and characterized by different techniques. The process has been tested in a fixed-bed reactor operated with feed composition in the range 5–60% for methane and 0–16% for oxygen. This way, the application of this process to many potential methane emissions (e.g., coal bed methane, natural gas leakages, landfill gas, anaerobic process emissions, etc.) is covered by this work. The performance of the process has been compared in terms of methanol yield and methane adsorption capacity. The conditions of the desorption step (type of sweep gas and temperature) have also been optimized to maximize the methanol yield.

## Materials and Methods

### Preparation of the Catalyst

The support of the catalyst is a commercial Na–mordenite (denoted as Na–MOR, Si/Al = 6.5) purchased from Zeolyst International. The method used for the preparation of the catalyst is based on the wet ion exchange in a 0.01 M copper(II) acetate solution at pH 5.7 (to avoid the undesired precipitation of copper hydroxides and maximize the concentration of partially hydrolyzed copper ions^41,48^). This solution was mixed with the zeolite (78 mL/g solid) and stirred overnight at room temperature. Then, the solid was filtered and washed. The whole process was repeated three times. After the last filtration, the resulting solid was dried overnight in an oven at 110 °C, pelletized, and sieved to a particle size in the range 0.355–1 mm. The catalyst is loaded into the reactor and activated at 450 °C (1 °C/min ramp) in a flow of air. This method was successfully used in a previous work^49^ and by other authors.^44,50^

### Characterization of the Catalyst

The X-ray powder diffraction (XRD) patterns of the catalyst samples were recorded on a Bruker D8 Discover diffractometer with a radiation scanning 2θ range of 5–55°. The quantification of the copper loading in the catalyst was done by dissolving a sample in aqua regia, followed by inductively coupled plasma mass spectrometry (ICP-MS) analysis.

The nitrogen adsorption and desorption isotherms of the materials were measured in a Micromeritics ASAP 2020 Plus apparatus at 77 K to obtain Brunauer–Emmett–Teller (BET) surface areas of the catalysts. Previously, the samples were degassed under vacuum at 150 °C for 10 h.

Temperature-programmed reduction (TPR) of the catalyst was performed in H_2_ using a Micromeritics AutoChem II 2920. A sample of 50 mg was introduced into a quartz tube and pretreated with a He stream at 200 °C for 2 h. After cooling down to room temperature, the sample was heated to 450 °C at 5 °C/min in a gas stream of 5% H_2_ in He. The concentration of H_2_ in the gas effluent was measured using an OmniStar GSD 301 mass spectrometer.

Ammonia temperature-programmed desorption (TPD) was also performed using the same equipment to observe the acidity of the catalyst and zeolite. First, the sample was saturated with NH_3_ for 1 h at room temperature. Then, the temperature was increased at a heating rate of 5 °C/min up to 450 °C to promote the desorption of NH_3_, which was monitored also by an OmniStar GSD 301 mass spectrometer.

A Thermo Nicolet Nexus spectrometer was used to perform the diffuse reflectance infrared Fourier transform spectroscopy analyses (DRIFTS). A total of 128 scans were used to obtain each spectrum. The spectrometer was equipped with a catalytic chamber with a ZnSe window for high-temperature treatment and interaction with the gas. The catalyst was activated in the chamber using an airflow (40 mL/min) at 450 °C for 2 h. The catalyst sample was contacted with methane (20% in He) and water vapor flows at reaction conditions.

### Experimental Device

The partial oxidation of CH_4_ into CH_3_OH was conducted in a stainless steel fixed-bed reactor (ID 6.8 mm, length 600 mm) placed in an electrical oven.^49^ The catalyst loading was 3 g, which corresponded to a bed length of 110 mm; the remaining reactor tube upstream of the catalyst bed was filled with glass spheres (1 mm). The gas flow inside the reactor tube was plug flow as indicated by the following relationships: ratio of the reactor ID to the catalyst particle size of at least 10 (10) and a ratio of bed length to catalyst particle size higher than 50 (162).^51^ These ratios ensure the correct distribution of the reactants and avoid the presence of preferential paths.

**Figure 1:**
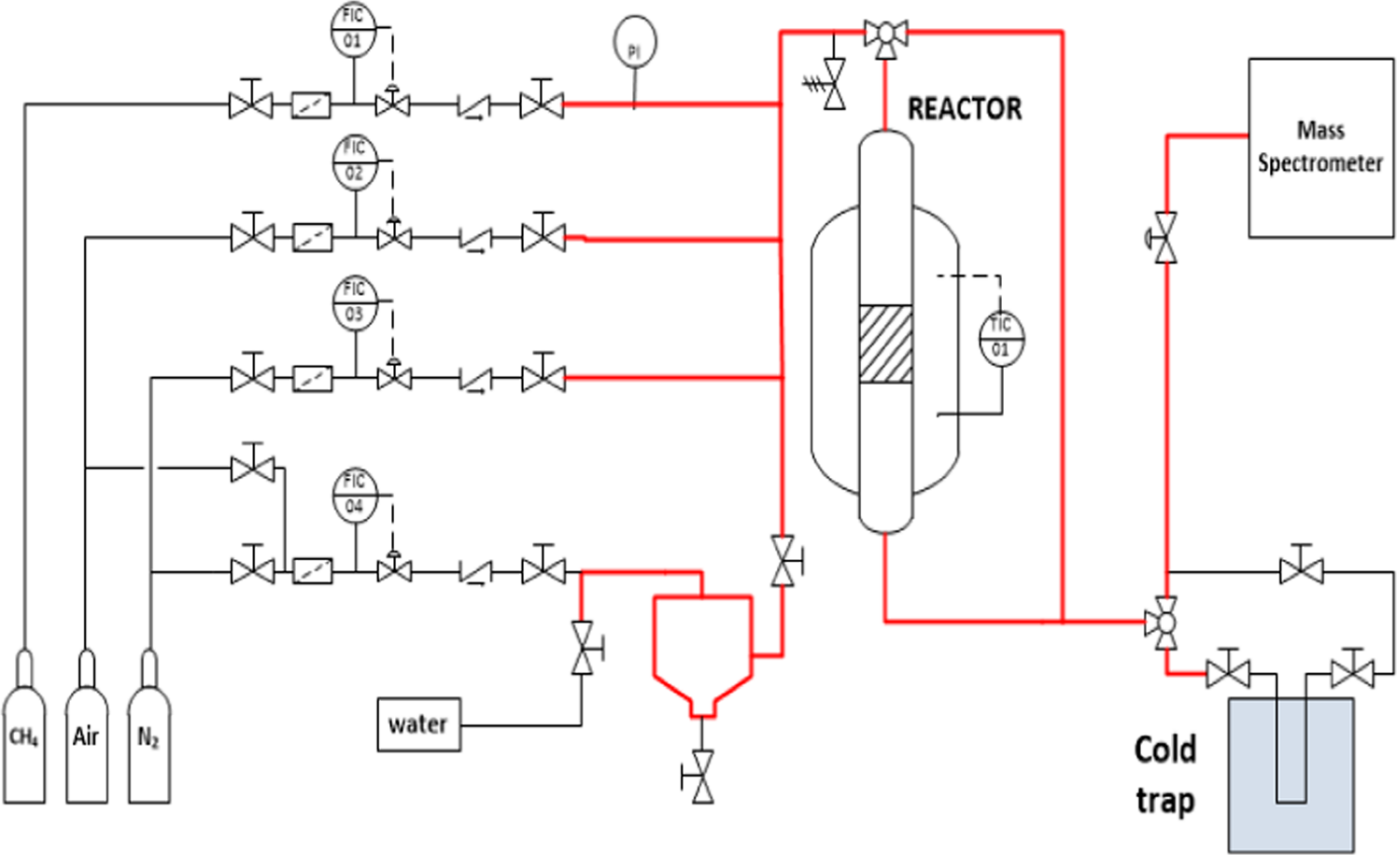
Scheme of the experimental device used. Red lines represent the pipes wrapped with heat tape.

The gases were supplied by Air Liquide in cylinders. The gas flow rates were set using Bronkhorst mass flow controllers; the desired concentration was obtained by mixing the gases in adequate proportions. In the desorption step, a water/gas stream is required. Water is introduced in the gas flow using a syringe pump. To ensure complete vaporization and prevent condensation, all the pipes were maintained at 150 °C using a heating tape. A scheme of the experimental rig is depicted in Figure 1.

The reactor effluent is analyzed online using a mass spectrometer (Omnistar GSD 301). During the desorption step, the reactor effluent is sent to a cold trap (at −50 °C) to condense species like methanol and water. The dry gas is analyzed in the mass spectrometer. The liquid sample obtained in this cold trap is analyzed in a gas chromatograph (Shimadzu GC-2010, CP-Sil 8CB column, flame ionization detector) and used to quantify the reaction yield.

### Reaction and Temperature-Programmed Oxidation Tests

The direct partial oxidation of methane to methanol is accomplished by a cyclic three-step process: adsorption, desorption, and activation. In between every step of the process, a purge with nitrogen (120 mL n.t.p./min) is used for 20 min to eliminate the remaining gases in the piping and bed voids. The purpose of this set of experiments was to test different methane (5–100%) and oxygen (0–16%) concentrations and study their influence on the performance of the catalyst. In the presence of air, methane is flammable in a range between 5 and 15%. However, oxygen–nitrogen–methane mixture is not flammable when oxygen concentrations are below 12%, regardless of the methane concentration. A concentration of 20% of CH_4_ was chosen to study the effect of the oxygen concentration on the performance of the catalyst since an oxygen concentration of 23% would be required to have a flammable mixture, 16% being the maximum oxygen concentration tested. The adsorption step was done at 200 °C by most of the authors since higher temperatures would barely increase the methanol yield^47^ and could promote the oxidation of methane to CO_2_. A temperature lower than 200 °C has a negative impact.^52^ A gas stream of 120 mL n.t.p./min (2.29 Nm^3^/(h kg_cat_)) was introduced in the reactor for 20 min during this stage.

For the desorption step, there are more differences in the conditions used by different authors. For this reason, temperatures between 150 and 200 °C were studied using air and nitrogen as carrier gas. This stage lasts for 4 h in a flow of 160 mL n.t.p./min with a 5.2% water in the carrier gas (3.04 Nm^3^/(h kg_cat_)). The flow rate is higher than that in the other steps to avoid water condensation in the pipes, which may produce discontinuities in the gas flow and concentration. After the desorption step, the reactor was cooled down and purged with nitrogen.

Catalyst activation is typically done using pure oxygen. The influence of oxygen partial pressure was studied in some works,^47^ and it was observed that pressures higher than 1 bar have a negative effect on the reaction yield. In the previous study,^49^ our group concluded that the use of air, instead of pure oxygen, is a better choice, increasing methanol production. In addition, the lower price of air also improves the economy in the scale-up of the process. The activation of the catalyst was done at a high temperature (ramp of 1 °C/min to 450 °C) since some authors have studied the influence of temperature at this stage, concluding that 450 °C is the optimal temperature when oxygen is the oxidizer.^50^ At this temperature, all of the water adsorbed on the catalyst is removed.^49,53^

Blank tests showed that no reaction takes place in the absence of catalyst or with the mordenite support.

Temperature-programmed oxidation (TPO) techniques are used to quantify the amount of methane adsorbed on the catalyst. Thus, after a regular adsorption step, the desorption step can be replaced by a TPO, in which a gas stream of synthetic air is introduced and, at the same time, the reactor temperature is increased to 450 °C (ramp of 10 °C/min). Methane adsorbed on the catalyst is desorbed and fully oxidized to CO_2_, which can be analyzed online using a mass spectrometer (signal with *m*/*z* = 44). This CO_2_ can be quantified using a calibration based on a TPO carried out on a sample of sodium bicarbonate.^34,44,45^ The conditions used in each step of both reaction and TPO tests are depicted in Table 1.

**Table 1:** Summary of the Conditions of Each Step for the Reaction and Temperature-Programmed Oxidation Tests

reaction tests	gas (mol %)	temperature (°C)	hold time (min)	GHSV (Nm^3^/(h kg_cat_))
adsorption	CH_4_/O_2_/N_2_	200	20	2.29
desorption	5.2 H_2_O/96.8 N_2_	150	240	3.04
activation^a^	20 O_2_/80 N_2_	450	240	2.29

TPO tests	gas (mol %)	temperature (°C)	hold time (min)	GHSV (Nm^3^/(h kg_cat_))
adsorption	CH_4_/O_2_/N_2_	200	20	2.29
TPO^b^	20 O_2_/80 N_2_	450		2.29
activation^a^	20 O_2_/80 N_2_	450	240	2.29

a Heating rate of 1 °C/min.

b Heating rate of 10 °C/min.

## Results and Discussion

### Catalyst Characterization

The ion-exchange procedure used for the preparation of the catalyst samples leads to zeolites with a copper loading of 4.5 wt %, according to the ICP-MS results. This copper loading is similar to the value reported by other authors using analogous preparation methodologies.^44^ It was reported that this copper concentration was stable, and no copper is lost after several reaction cycles.

**Figure 2:**
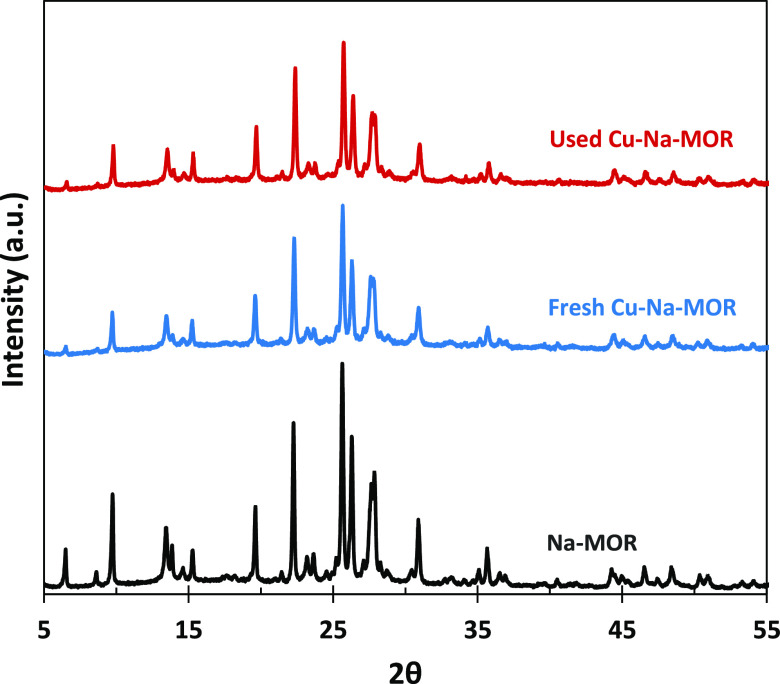
XRD patterns of Na–MOR and fresh and used Cu–Na–MOR.

The XRD spectra shown in Figure 2 indicate that both Na–MOR and Cu–Na–MOR exhibit the characteristic peaks of the MOR crystal structure. The intensity of the peaks is lower after the ion exchange, suggesting that the whole preparation process slightly affects the crystallinity of the sample, which according to the Scherrer equation is 20% lower. No new crystalline phases were detected in the Cu–Na–MOR samples. This indicates that there are no copper or copper oxide crystalline particles with a diameter above 2 or 3 nm.^29,34^Figure 2 also shows that the MOR structure is stable after being subjected to several reactions and TPO tests, indicating good structural stability of the catalyst.

In the nitrogen physisorption tests, type I isotherms are obtained, indicating that this is a microporous material with relatively small external surface and narrow micropores (of width <1 nm).^54^ The BET surface areas of the materials have been obtained from nitrogen adsorption/desorption tests, being 376 m^2^/g for Na–MOR and 359 m^2^/g for Cu–Na–MOR. This small reduction in the surface area of the materials can be explained due to the blockage of some pores with copper oxide particles.^55^ A similar value of 355 m^2^/g was obtained for a used Cu–Na–MOR sample, which reinforces the idea of the good structural stability of the material.

**Figure 3:**
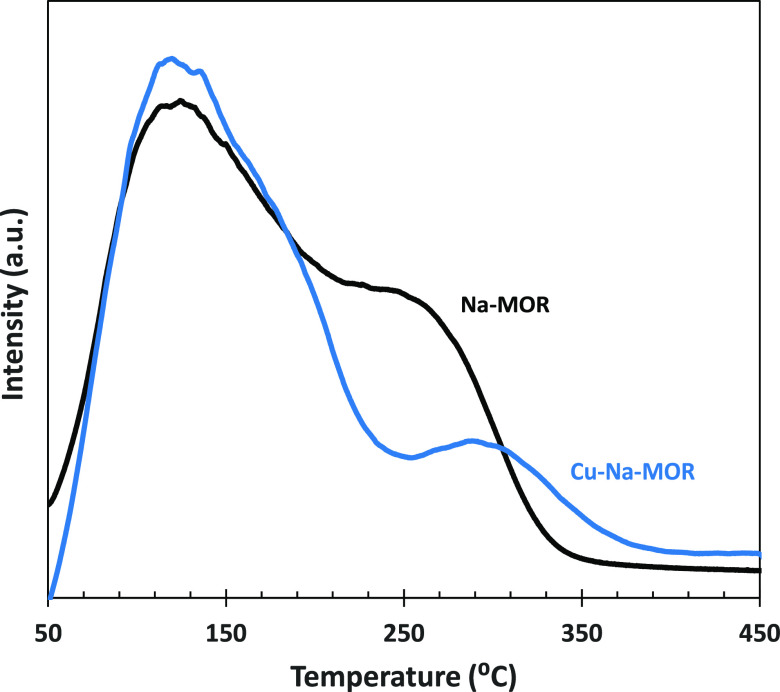
NH_3_-TPD patterns of Na–MOR and Cu–Na–MOR (activated).

Ammonia temperature-programmed desorption (NH_3_-TPD) tests (Figure 3) were used to measure the acidity of the Na–MOR support and the activated Cu–Na–MOR catalyst. Two peaks are observed at low temperatures, corresponding to weak acid centers of the zeolite surface.^56^ The presence of two peaks can be related to two types of channels of the zeolite structure. The larger 12MR channels are responsible for the peak observed at 150 °C. The smaller 8MR pockets lead to a peak at 275 °C because ammonia desorption is more difficult from these channels and a higher temperature is required. The Cu–Na–MOR catalyst shows a very similar NH_3_-TPD pattern, but the high-temperature peak decreases in intensity and shifts to a higher temperature.

The transmission electron microscopy (TEM) images of this catalyst showed copper aggregates of two sizes, 8–18 and 1.4–2.8 nm.^49^ These copper particles could be responsible for blocking part of the smaller 8MR channels of the zeolite structure, which would agree with the decrease in intensity of the high-temperature peak of NH_3_-TPD.

The H_2_-TPR test was performed on an activated Cu–Na–MOR catalyst sample. Only a single peak related to hydrogen consumption was observed at 180 °C, attributed to the reduction of the most accessible copper clusters. The temperature of this reduction is lower than that observed for reference copper oxides due to the small size of the copper clusters and their dispersion on the zeolite structure.^34^

**Figure 4:**
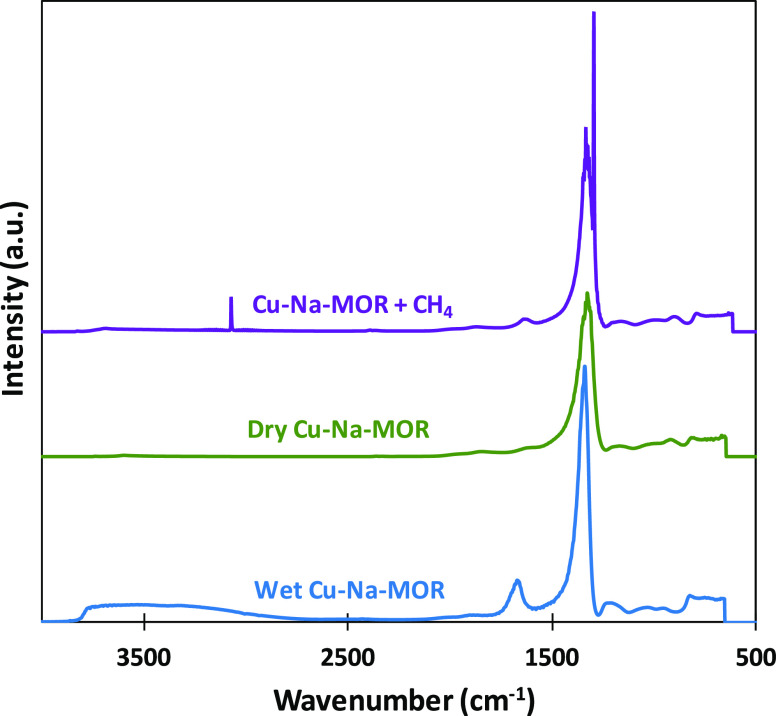
DRIFTS analysis of activated Cu–Na–MOR at reaction conditions: dry, wet, and methane adsorption.

Diffuse reflectance infrared Fourier transform spectroscopy (DRIFT) analysis of the activated Cu–Na–MOR catalyst is depicted in Figure 4. A large peak was observed at 1350 cm^–1^, which was also reported for the Na–MOR support, so it is attributed to the characteristic of the zeolite structure. The small peaks observed between 1000 and 500 cm^–1^ might be related to the O–O and Cu–O bonds of the active centers.^57^ The introduction of water produces two new peaks: a broadband between 4000 and 3000 cm^–1^, related to the stretching vibrations of water, and a peak at 1600 cm^–1^, caused by bending vibrations.^58^ These peaks disappeared when the catalyst was exposed to an airstream at 450 °C, which confirmed that the regenerating conditions were enough to fully remove water from the zeolite structure.

Finally, a methane flow (20% CH_4_ in He) was introduced into the chamber at 200 °C. Two new sharp peaks were observed at 3000 and 1300 cm^–1^, attributed to the C–H bond stretching and bending vibrations.^59^ These peaks disappeared very quickly when the temperature was increased to only 250 °C, which suggested that methane was weakly bonded to the catalyst. It should be noted that the DRIFTS analysis is a superficial technique, and only outer or exposed interactions can be recorded. Hence, it is difficult to measure the interactions inside the microporous channels of the zeolite structure, which are responsible for methane activation in the partial oxidation to methanol. The observed peaks are attributed to the adsorption sites of the copper particles observed in the TEM images, placed outside the zeolite channels. In these sites, the confinement effects of the zeolite structure are not detected and, for this reason, the observed interactions are weak.

### Preliminary Tests

#### Measurement of Catalyst Performance

Preliminary studies were performed at an adsorption temperature of 200 °C using pure methane as a source gas to evaluate the behavior of the catalyst and as a reference for the following experiments. The production of methanol during the desorption step, performed at 150 °C with a wet nitrogen stream, was 164 μmol/g Cu. After the desorption step, the catalyst was regenerated in air and, in these conditions, 100–300 μmol CO_2_/g Cu were detected in the effluent. This suggests that some methane remained adsorbed on the catalyst even after 4 h of desorption and was only released from the catalyst, as CO_2_, when high temperature and oxidizing conditions were applied.

To assess the performance of the catalyst, the adsorption capacity was evaluated by means of TPO tests. In these tests, two peaks are identified: one at 230 °C and another one close to 300 °C, suggesting that there are two types of adsorption sites for methane on the catalyst surface. The high-temperature peak is attributed to stronger methane adsorption. The total amount of methane adsorbed was 2041 μmol/g Cu, of which 38% corresponds to the low-temperature peak and 62% to the high-temperature one. These tests indicate that only a small fraction of the adsorbed methane (8.0%) can react to produce methanol.

The catalyst was stable during all of the experimental programs, as periodically checked in control tests.

### Optimization of the Desorption Step

To simplify the overall process, the use of the same temperature in the adsorption and desorption steps would be preferable. It is well-known that decreasing or increasing the adsorption temperature (200 °C) has a large negative impact on the methanol yield.^52^ For this reason, the temperature of the adsorption step has been set to 200 °C, while the conditions of the desorption step have been optimized.

**Figure 5:**
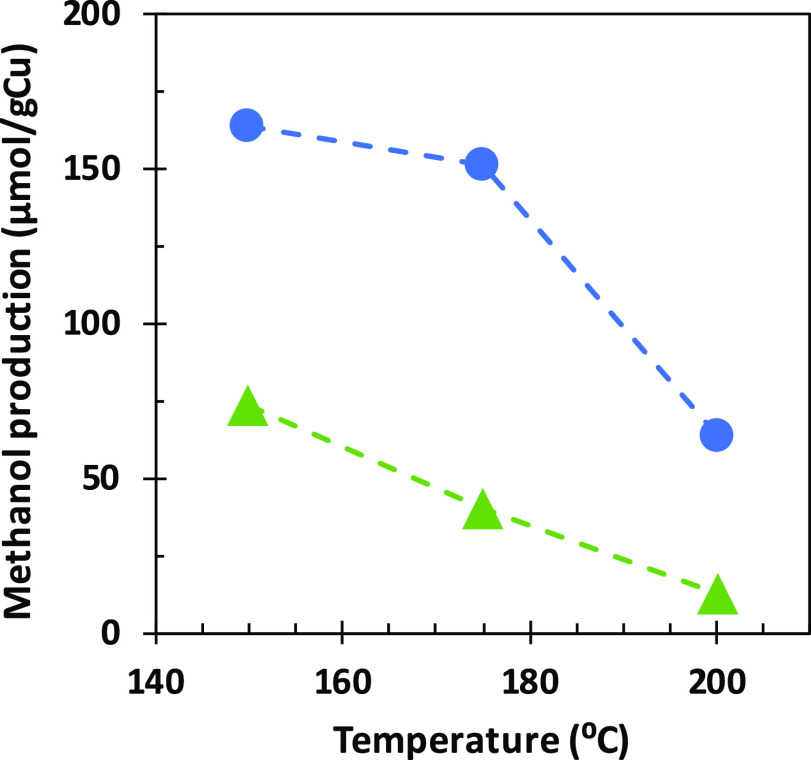
Optimization of the desorption step: effect of temperature and carrier gas on methanol production: N_2_, blue circle solid; air, green triangle up solid.

The results are summarized in Figure 5 in terms of methanol production. Methanol productivity decreased on increasing the desorption temperature. This is due to a higher fraction of the adsorbed methane being oxidized to CO_2_ at higher temperatures. Hence, it can be concluded that the temperature of the desorption step is also a critical parameter and should be carefully controlled. The gas flow is formed by water vapor in a carrier gas. In a previous study,^49^ the optimum gas flow rate and water composition were determined to be 160 mL n.t.p./min and 5.2%, respectively. In that work, nitrogen was used as the carrier gas. In the present work, nitrogen carrier gas has been replaced by air, as depicted in Figure 5. As shown, the use of air reduces the production of methanol considerably from 164 μmol/g Cu to 74 μmol/g Cu in the test at 150 °C (a reduction of 55%). At the worst conditions, i.e., air at 200 °C, the production of methanol is reduced to only 13 μmol/g Cu. Considering these results, the best option to maximize methanol production is the use of nitrogen as carrier gas at 150 °C.

### Application to Lean Methane Feedstocks

#### Influence of Methane Concentration

The influence of methane has been studied in the range 5–100% and using nitrogen as a balance gas (to prevent any side effect caused by other secondary molecules). The tests have been done at the conditions determined in the preliminary tests, with the desorption temperature of 150 °C and using nitrogen as the desorption carrier gas.

**Figure 6:**
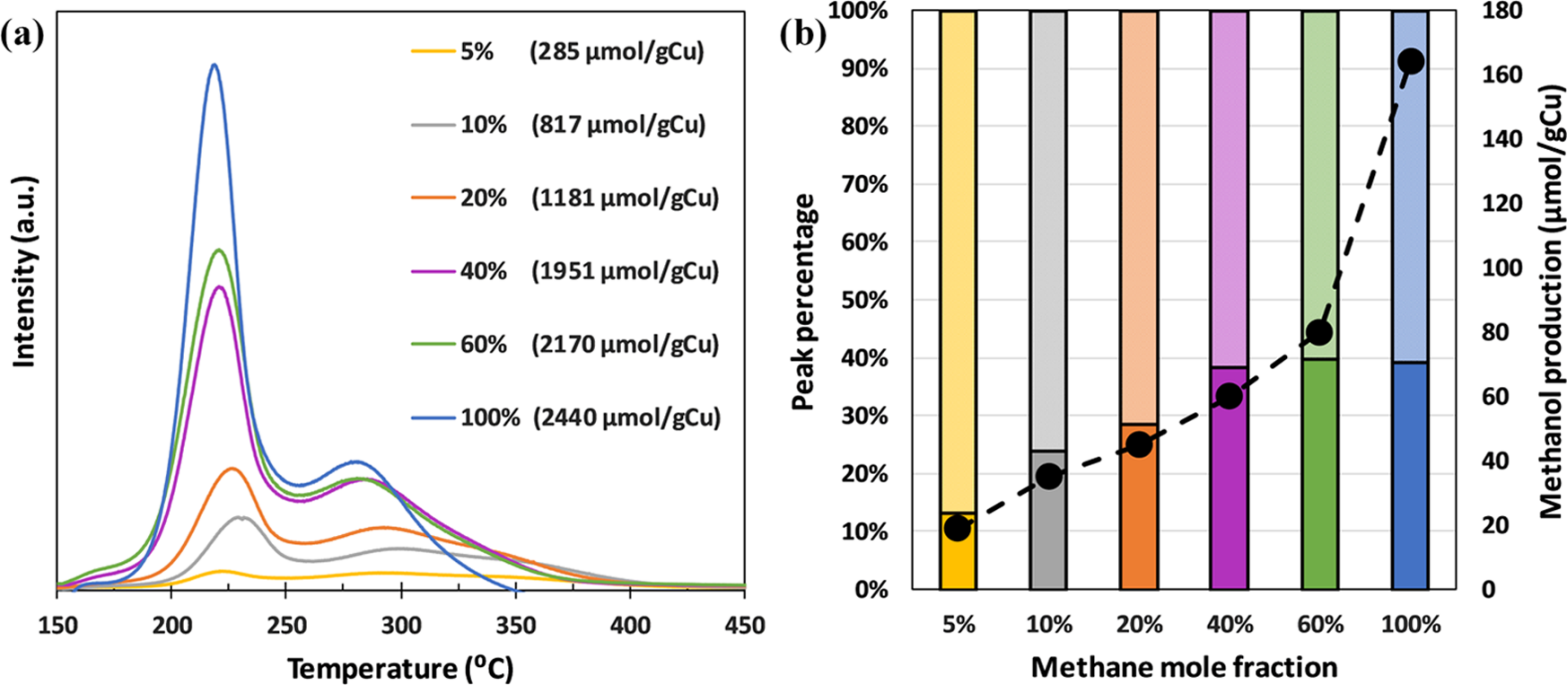
(a) MS signal attributed to CO_2_ (*m*/*z* = 44) obtained in the TPO tests carried out after the methane adsorption step at 200 °C and different methane mole fractions in nitrogen. (b) Distribution of methane adsorbed as a function of methane mole fraction (filled bars correspond to the low-temperature TPO peak and checked bars to the high-temperature one). Methanol production in the reaction step (●) as a function of methane mole fraction.

The methane adsorption capacity has been evaluated using TPO tests, as shown in Figure 6a. As the methane concentration decreases, the amount of methane adsorbed on the catalyst also decreases, with a minimum adsorption value of 285 μmol/g Cu at 5% methane (this is a decrease of 88% with respect to pure methane). The amount of methane adsorbed in the two peaks of the TPO of Figure 6a has been quantified separately, as depicted in Figure 6b. Thus, for lower methane concentrations, the relative importance of the high-temperature peak increases, with a maximum contribution to the total amount adsorbed of 87% at 5% methane. This reinforces the hypothesis of methane activation in the partial oxidation to methanol being associated with the strong adsorption of methane owing to the high-temperature peak of the TPO test. Thereby, methane, which is strongly linked to the active sites, may be affected to a lower extent by the decrease in methane gas partial pressure. As observed in Figure 6a, the amount of methane adsorbed increases on increasing the methane mole fraction and the same trend is observed for methanol production (Figure 6b). For pure methane, a methanol production of 164 μmol/g Cu was obtained, while the production decreases to 19 μmol/g Cu with 5% methane (similar to a decrease of 88% observed for methane adsorption).

### Influence of Oxygen Concentration

**Figure 7:**
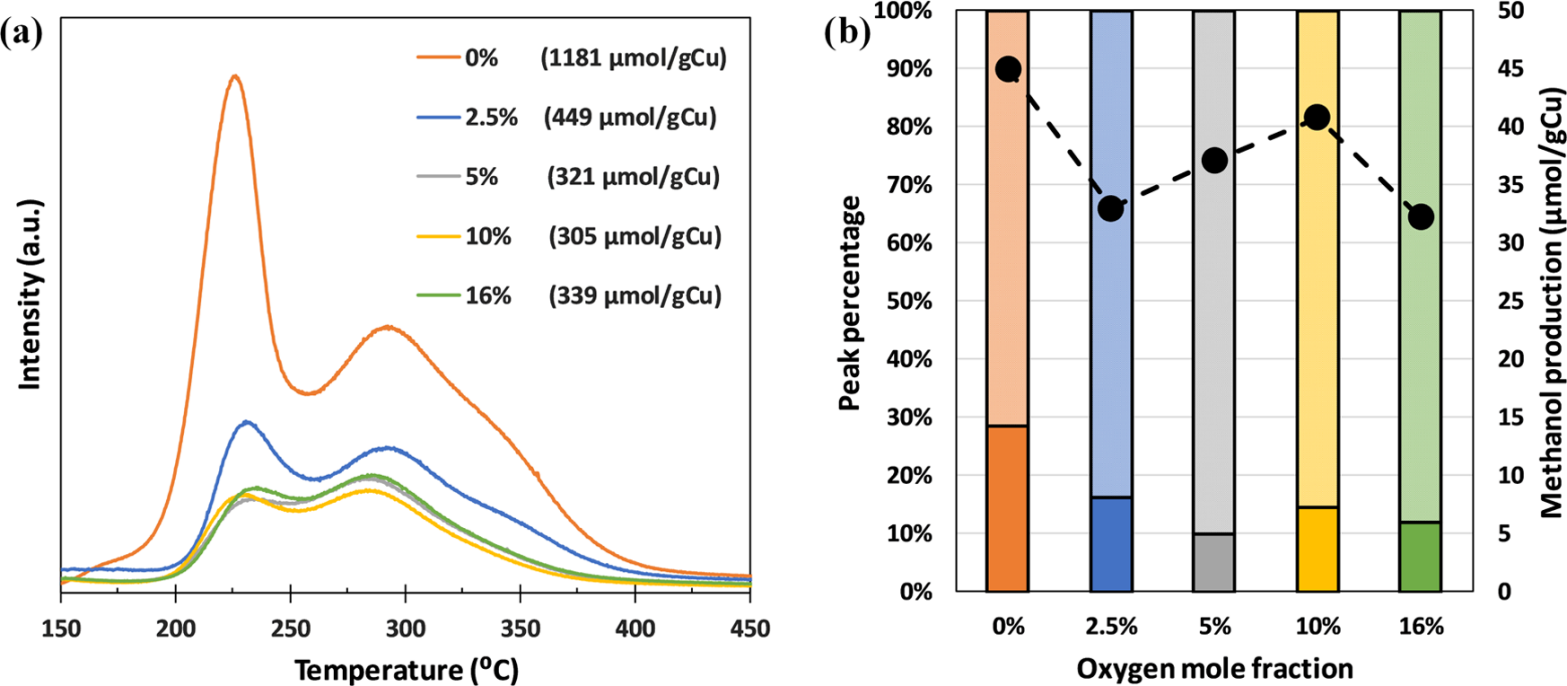
(a) MS signal attributed to CO_2_ (*m*/*z* = 44) obtained in the TPO tests carried out after the methane adsorption step at 200 °C, a methane concentration of 20%, and different oxygen mole fractions. (b) Distribution of methane adsorbed as a function of oxygen mole fraction (filled bars correspond to the low-temperature TPO peak and checked bars to the high-temperature one). Methanol production in the reaction step (●) as a function of oxygen mole fraction.

Oxygen can be present in many lean methane feedstocks, e.g., due to air intrusion during the generation or capture of the methane source. This oxidant may have a negative influence during the adsorption step of the process, and for this reason, additional experiments have been proposed. The same methodology explained before has been followed but using a feed made of methane, oxygen, and nitrogen in different proportions. First, a 20% methane mixture with an oxygen mole fraction in the range of 2.5–16% has been studied. Figure 7a summarizes the results of the TPO tests. It is clearly observed that the presence of oxygen in the gas feed has a negative effect on methane adsorption, which decreases from 1181 μmol/g Cu (in the absence of O_2_) to 339 μmol/g Cu for an oxygen concentration of 16%. The TPO tests also show that the amount of adsorbed methane remains practically constant (305–339 μmol/g Cu) for oxygen mole fractions higher than 5%. For 2.5% O_2_, methane adsorption is slightly higher, 449 μmol/g Cu. These results indicate that even the lowest oxygen concentration has a huge impact on the adsorption capacity of the catalyst. The role of oxygen is explained by promoting the complete oxidation of part of the adsorbed methane to CO_2_. These oxidizing conditions may even promote the re-oxidation of some weak active centers of the catalyst, which may continuously turn methane into CO_2_.

However, the reaction experiments, carried out after an adsorption step in the presence of oxygen, showed that the decrease in methanol production was lower than that observed in methane adsorption (Figure 7b). Methanol production was 45 μmol/g Cu in the absence of oxygen and decreased to 32 μmol/g Cu in the case of 16% oxygen (the harshest conditions). This is a reduction of 29% in methanol productivity, which is far from the 74% reduction in methane adsorption capacity measured during the TPO tests.

These results can be explained by the presence of different types of active sites on the catalyst surface. Thus, most of the methane that is oxidized to CO_2_ during the adsorption step would have been adsorbed on centers that are not able to catalyze the partial oxidation to methanol. This is shown in Figure 7b, in terms of the relative contribution of the two peaks appearing in the TPO tests. When oxygen was introduced in the adsorption step, an increase in the percentage contribution of the high-temperature peak, i.e., that associated with stronger methane adsorption, is observed (from 71% to a range between 83 and 90%). Conversely, the peak associated with weakly bonded methane (the low-temperature one) is strongly affected by the presence of oxygen, decreasing its relative contribution to the total methane adsorption capacity.

Additional tests for other methane concentrations were done and their results, compared to a 20% methane feed, are displayed in Table 2. It can be observed that there is a reduction of 75% in methane adsorption capacity and 33% in methanol productivity in the case of a 40% methane feedstock. For a lower methane feed concentration (10%), the reduction in the amount of methane adsorbed is similar (77%) while methanol productivity barely decreased (9%). These results agree with those of the previous experiments, discussed before. The effect of oxygen on methane adsorption capacity is similar for all methane concentrations; percentage reduction being close. On the contrary, methanol production in the presence of oxygen is less affected by the presence of oxygen, especially when lower methane concentrations are tested. This is an advantage of this process when used for the upgrading of lean methane feedstocks containing oxygen.

**Table 2:** Summary of Additional Reactions Performed with the Same Oxygen Concentrations but Different Methane Mole Fraction^a^

methane fraction (mol %)	oxygen fraction (mol %)	methane adsorption (μmol/g Cu)	methanol production (μmol/g Cu)
10	0	817	35
	10	234 (71%)	32 (9%)
20	0	1181	45
	10	305 (74%)	41 (9%)
40	0	1951	60
	10	495 (75%)	40 (33%)

a Percentage reductions are indicated in parenthesis.

## Conclusions

The direct partial oxidation of methane to methanol over a Cu–mordenite catalyst has been studied in a fixed-bed continuous reactor. The reaction has been accomplished by a chemical looping process made of three steps: adsorption, desorption, and regeneration. Many methane feedstocks are diluted in methane or contaminated with oxygen. The experiments have been aimed at evaluating the influence of these two variables on the process performance.

A lower methane feed concentration in the adsorption step led to a lower amount of adsorbed methane (as indirectly measured in the TPO tests) and lower methanol productivity. Two methane adsorption centers of different strengths were identified on the catalyst surface. The one with a higher strength (i.e., with a higher release temperature in the TPO tests) and associated with methanol formation was less prone to a reduction in the methane partial pressure. Although methane preconcentration will increase methanol productivities, we have demonstrated that dilute methane feedstocks can be used as raw material for this reaction.

The presence of oxygen in the feed of the adsorption step had a strong negative influence on the amount of adsorbed methane. However, methanol productivity was affected only slightly (e.g., a feed gas of 20% methane and 10% oxygen showed a decrease of 74% in methane adsorption and only 9% in methanol production). According to this, it can be concluded that methane adsorption on the active centers capable of transforming methane into methanol is not affected by the presence of oxygen. This is an important outcome since many methane feedstocks are contaminated with oxygen.
